# Experimental Study of Reinforced Concrete T-Beam Retrofitted with Ultra-High-Performance Concrete under Cyclic and Ultimate Flexural Loading

**DOI:** 10.3390/ma16247595

**Published:** 2023-12-11

**Authors:** Abbas Khodayari, Sheharyar Rehmat, Alireza Valikhani, Atorod Azizinamini

**Affiliations:** 1 Department of Civil and Environmental Engineering, Florida International University, Miami, FL 33174, USA; aazizina@fiu.edu; 2 TYLin, Olympia, WA 98005, USA; 3 Genex Systems/Turner-Fairbank Highway Research Center, 6300 Georgetown Pike, Mclean, VA 22101, USA; a.valikhani.ctr@dot.gov

**Keywords:** beam retrofit, ultra-high-performance concrete (UHPC), flexural strength, cyclic loading, accelerated retrofit solution, failure mode

## Abstract

Structurally deficient bridges are commonly retrofitted using conventional methodologies, including reinforced concrete, steel jackets, and fiber-reinforced polymers. Although these retrofit methods aim to improve structural performance, exposure to aggressive environments may undermine the durability performance of the retrofit material. More recently, ultra-high-performance concrete (UHPC) has provided an alternative to conventional construction methods, with its superior material characteristics favoring its use in retrofit applications. In this study, a large-scale reinforced concrete (RC) T-beam is constructed and artificially damaged. The T-beam is then retrofitted with an external envelope of UHPC on all faces. Sandblasting is introduced to the surface, providing partially exposed reinforcement in the T-beam to simulate material deterioration. Additional reinforcement is placed in the web and flange, followed by casting the enveloping layer of UHPC around the specimen. The feasibility of this method is discussed, and the structural performance of the beam is assessed by subjecting the beam to cyclic and ultimate flexural loading. This paper presents the results of cyclic and ultimate testing on the RC-UHPC composite T-beam regarding load–displacement, failure mode, and strain responses. The retrofitted T-beam specimen is subjected to a cyclic loading range of 131 kN for 1.576 million cycles. Despite no visible cracks in the cyclic testing, the specimen experiences a 12.22% degradation in stiffness. During the ultimate flexural testing, the specimen shows no relative slip between the two concretes, and the typical flexural failure mode is observed. By increasing the longitudinal reinforcement ratio in the web, the failure mode can shift from localized cracking, predominantly observed in the UHPC shell, toward a more distributed cracking pattern along the length of the beam, which is similar to conventional reinforced concrete beams.

## 1. Introduction

The exposure of bridge elements to deleterious chemicals and deicing salts can facilitate deterioration and reduce the service life of reinforced concrete (RC) bridges. In addition to the exposure to severe environmental conditions, bridges also undergo fatigue loading, which can further exacerbate these deterioration issues. Efforts have been devoted to developing and implementing various materials for repairing and retrofitting, including concrete, steel, and fiber-reinforced polymer jackets, to mitigate the actions of the two deterioration factors [1,2,3]. However, the long-term performance of these retrofit materials and methods has shown recurring durability issues or incompatibility with substrate-damaged concrete [4]. Additionally, the process of repair and retrofitting leads to high costs and can be time-consuming; therefore, it is desirable to develop innovative materials that will prolong the service life of the bridge member by delaying the penetration of aggressive chemicals and that are less sensitive to the substrate conditions. Although the primary objective of any retrofit initiative is to contribute toward the increase in structural demand, the protection of the existing structure needs to be taken into account as well. Hence, there is a need to develop and explore innovative structural materials suited for retrofit needs and the prolonged service life of the member.

Several research studies have recently experimentally tested ultra-high-performance concrete (UHPC) as an alternative to conventional retrofit methods for deficient structures [5,6,7]. Compared to normal-strength concrete (NSC), the enhanced mechanical and material properties of UHPC are a result of the low water-to-binder ratio, the optimized gradation of constituents, and the presence of discontinuous internal fibers. The material benefits from low permeability and negligible chloride penetration and exhibits high compressive and tensile strength, owing to the dense microstructure [8]. Due to its rheological properties, UHPC allows the design and construction of constricted forms and complex geometries common for retrofit applications. These advantages of UHPC also lend themselves to remedial applications for structural members by providing an external protective layer against environmentally severe influences. Besides attenuating the effects of aggressive environments, UHPC can also help to reduce the impact stresses imparted by extreme and continuous loading [9].

UHPC has gained attention in the United States in the past few years, particularly for constructing new bridge elements [10,11,12,13,14]. Several studies have documented the implementation methods and performances of these elements [15]. However, the current commercial cost of most of the proprietary UHPC mixes is significantly larger than NSC, which leads to an uneconomical solution for new construction. Hence, the idea is not to use the material for new structural members but to strengthen the existing ones with extensive damage. Several research endeavors have focused on implementing UHPC as the retrofit material for reinforced beams, columns, and beam–column joints [16,17,18,19]. However, the modalities of field implementation and best practices of repair and strengthening have not been fully developed and, consequently, remain largely unimplemented for structurally deficient bridges.

## 2. Previous Research

Several studies have been conducted to understand the structural performance of UHPC as a retrofit material, proving its durability improvement and highlighting the structural enhancements in the retrofitted section. Safdar et al. [20] have implemented ultra-high-performance fiber-reinforced concrete (UHPFRC) layers with varying depths to retrofit reinforced concrete beams in both compression and tension zones. Their results indicated that increasing the thickness of the top and bottom retrofit zone improved the ultimate capacity to the order of 1.2 to 1.31 compared to conventional reinforced control beams. Zhang et al. [21] conducted an experimental study on damaged scaled bridge decks strengthened with UHPC. The results showed that UHPC incorporation on the tension side significantly improved the ultimate capacity. In addition, UHPC was incorporated on the compression side, resulting in an increase of 30 percent in ultimate capacity. The three-side jacketing of UHPC of rectangular beams was studied by Al-Osta et al. [22] and yielded the highest enhancement in ultimate load and stiffness when compared to the bottom and side retrofitting techniques. The improvement was assigned to the increased moment of inertia under three-sided retrofitting, with the additional tension engaged from the sides of the T-beam. Zhang et al. [23] carried out UHPC strengthening of reinforced concrete beams, which were preloaded to induce varying degrees of damage. The flexural testing on the beams that were strengthened on their tensile zone showed that the ultimate load-carrying capacity of the beams was inversely proportional to the degree of pre-damages in the beam. Furthermore, reinforcing the UHPC layer with a steel mesh significantly improved the cracking and ultimate load capacity of pre-damaged beams.

## 3. Objective

This research aims to investigate the effectiveness of UHPC encapsulation in improving concrete T-beam performance and service life. The use of ultra-high-performance concrete (UHPC) in structural retrofitting has gained increasing attention due to its numerous advantages over traditional concrete. UHPC has a compressive strength of up to 200 MPa, significantly higher than the typical 30–40 MPa of normal-strength concrete (NSC). This high strength increases the load-carrying capacity and improves retrofitted structure durability.

In addition, UHPC has a much lower porosity than NSC, which results in a denser and more impermeable material. This makes UHPC more resistant to corrosion and chemical attack, which can be particularly beneficial in harsh environments such as bridges exposed to saltwater or road deicing salts. With its exceptional flowability, UHPC can easily flow into cracks and voids in the existing structure, resulting in a strong bond with the underlying concrete substrate. The high bond strength of UHPC is another key advantage, as it can enhance the structural performance of the retrofit system by distributing stresses more evenly across the interface between UHPC and NSC. This helps to prevent the debonding and delamination of the UHPC layer, which can compromise the effectiveness of the retrofit.

Shrinkage is a common concern when using traditional concrete materials, but UHPC has minimal shrinkage due to its low water-to-cement ratio and the addition of fibers [16]. Additionally, the autogenous shrinkage characteristic of UHPC can be further controlled by employing an appropriate heat treatment. UHPC also has a low coefficient of thermal expansion. As a result, the material is less susceptible to cracking from temperature changes, even when used with NSC substrates, which have different thermal properties. In addition, the low coefficient of thermal expansion of UHPC helps to maintain the composite action between the UHPC overlay and the NSC substrate, preventing any delamination or separation at the interface.

Using UHPC as a retrofit material can provide an aesthetically pleasing appearance with a smooth surface that enhances the overall visual appeal of the structure. This is possible due to the high flowability of UHPC, which allows it to replicate the texture of the formwork used during casting.

The project timeline involved multiple stages, starting with material and component testing before the experimental study of the T-beam specimen [24,25]. These tests evaluated the UHPC material properties, flow characteristics, and interface behavior between the UHPC shell and normal-strength concrete substrate. Subsequently, interface bond tests were conducted to evaluate the bond strength between UHPC and substrate concrete. The findings of these tests demonstrated that a combination of sandblasted surfaces with mechanical connectors provided the maximum bond strength between the UHPC and the substrate. In contrast, bonding agents did not perform well regarding bond strength. Following this, the retrofitting plan for the T-beam specimen was finalized, and the fabrication process began.

## 4. Overview of Methodology

This study selected a rational section to provide a realistic representation of T-girders commonly used in bridges. By replicating a real-life girder section, this study aimed to generate relevant findings and insights that can be applied to actual bridge repair and retrofit projects using UHPC.

The initial reinforced concrete section was transformed into a damaged section by simulating damages in the concrete cover and partially exposing both longitudinal and transverse reinforcement in the web and flange. Although these artificially induced damages are not emulative of actual material characteristics of a deteriorated beam, the assumption was made that the lack of cover and reduced section will adequately represent a structurally deficient section.

The reduced beam section was then preloaded to simulate cracks under service loads. Mechanical connectors were installed onto the post-sandblasted surfaces to improve the performance of the interface. A new retrofit methodology was proposed for a T-beam in which all exposed faces of the structural member were encased in UHPC. Additional reinforcement was placed in the web and flange before casting the enveloping UHPC layer around the T-beam. Finally, the T-beam was covered in plastic sheets and allowed to cure for ten days.

In order to evaluate the effectiveness of the retrofit approach, moment–curvature analyses were performed on both damaged and retrofitted T-beam sections. Subsequently, as a proof of concept, an experimental study was conducted to confirm the viability of the proposed retrofit methodology. Finally, the results of the experimental study were compared to the numerical data obtained from the moment–curvature analysis, which verified the validity of the analysis. This comprehensive analysis and experimentation provided valuable insights into the potential of UHPC for repairing and retrofitting existing bridge structures.

## 5. Experimental Program

### 5.1. T-Beam Specimen

**Figure 1:**
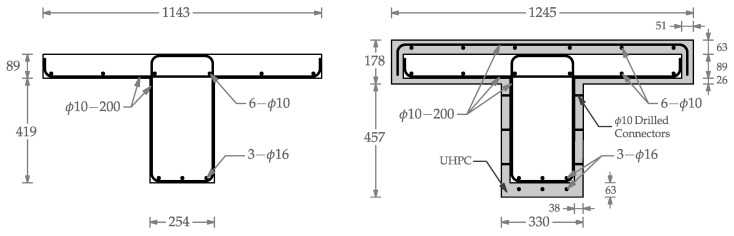
Details of the damaged and retrofitted T-beam specimen (unit: mm).

The initial damaged T-beam featured a flange with dimensions of 1143 × 89 mm (45 × 3.5 in) and a web measuring 254 × 419 mm (10 × 16.5 in). The web of the beam incorporated three ϕ16 tension reinforcements within the web, each having a clear cover of 12.5 mm (0.5 in). Additionally, closed ϕ10 stirrups were positioned at 200 mm intervals. The flange reinforcement comprised six ϕ10 bars at the bottom of the flange, along with single-legged ϕ10 shear reinforcement spaced at 200 mm intervals. Since the depth of the beam did not exceed 91.4 cm (36 in), no skin reinforcement was required [26]. For the retrofitted beam, three additional ϕ16 tension reinforcements and six ϕ10 reinforcements were placed at the bottom of the web and on the top of the flange, respectively. Figure 1 shows the dimensions and reinforcement details for the damaged and retrofitted T-beam.

### 5.2. Moment–Curvature Analysis

Moment–curvature analyses were conducted to determine the flexural capacity of the damaged and retrofitted sections. The calculations were based on the mechanical properties of steel and concrete materials obtained during the material testing study [24,25].

For all ASTM A615 Grade 60 steel reinforcements in the sections, elastic–perfectly plastic behavior was assumed ($F_{y}=478\;\mathrm{MPa}$, $E_{s}=2\times 10^{5}\;\mathrm{MPa}$). The stress–strain responses for normal-strength concrete (NSC) were derived from the model proposed by Mander et al. [27]. The compressive strength of NSC was 39.9 MPa, and the concrete crushing strain was taken as 0.003. The tensile strength of NSC was not considered in these calculations since the initial section was preloaded to a value of 97.9 kN, inducing a moment of 104.5 kNm in the middle of the specimen. This preloading led to a mid-span deflection of 2.3 mm and the emergence of hairline cracks at the bottom of the web, spreading over a distance of 1295 mm, thereby simulating damages under service loads.

**Figure 2:**
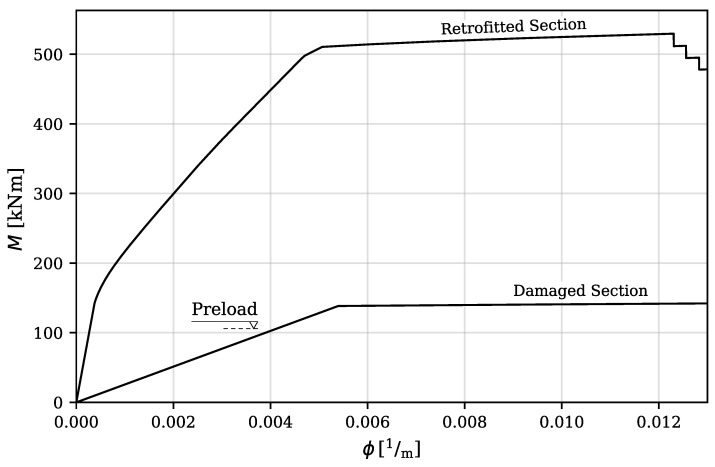
Moment–curvature responses of the damaged and retrofitted T-beam sections.

For the simulation of UHPC, a multilinear curve was defined to represent the stress–strain response of UHPC. The compressive strength of UHPC was determined to be 126 MPa based on the results of compressive testing on cylinder specimens following ASTM Standard C39/C39M [28]. The Young’s modulus for UHPC was calculated, using the formula $E_{c}=4069\sqrt{f_{c}^{\prime }}$ (in MPa units), as proposed by Graybeal and Stone [29], as 45,700 MPa. The softening region for UHPC under compression was modeled using a linear curve, reaching zero stress at a strain value of 0.007. The tensile strength of UHPC was assumed to maintain a constant value of 7.27 MPa until strain values of 0.007, at which point it diminished to zero. The bending moment capacities of the damaged and retrofitted sections were estimated to be 155 kNm and 529 kNm, respectively. A comparison of the moment–curvature responses is presented in Figure 2.

### 5.3. Construction

**Figure 3:**
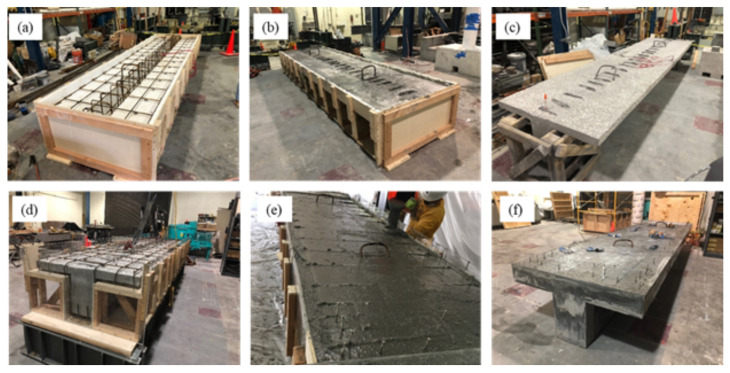
Construction of the T-beam specimen: (**a**) formwork for normal concrete; (**b**) casting of the concrete specimen; (**c**) reduced section after sandblasting; (**d**) placement of additional reinforcement; (**e**) UHPC casting; (**f**) completed section.

The first step of construction was casting the reduced core of the specimen. Once forms were stripped, the surface was sandblasted. Next, additional reinforcement was placed outside the core, and the mechanical connectors were affixed. Finally, the UHPC layer was cast. After casting, the specimen was cured for ten days using a wet burlap. Figure 3 shows photos from the various stages of the construction sequence. Additional details of the construction process are provided in the following paragraphs.

The bond performance was enhanced by sandblasting all surfaces of the reduced core, resulting in 4–6 mm surface indentations corresponding to the depth of concrete removed. Sandblasting was performed with moderate pressure to ensure adequate roughening while protecting the shear reinforcement. The shear reinforcement of the web was partially exposed during sandblasting, conducted at a distance of approximately 20 cm using a medium-grain-size abrasive material to avoid abrading the reinforcing bars. After sandblasting, the exposed surfaces were cleaned of any loose particles using a high-pressure water jet. Mechanical connectors consisting of ϕ10 reinforcing bars with a length of 38.1 mm (1.5 in) were then embedded to a depth of 12.7 mm (0.5 in) into the beam surfaces to improve the performance of the interface. The connectors were placed in a rhombus pattern on the top of the flange, sides, and bottom of the web. The connectors were affixed in the drilled cavities with an adhesive, and a protruding length of 25.4 mm (1 in) became embedded in the UHPC.

The specimen geometry and the volume of UHPC needed for encapsulation posed laboratory implementation challenges. Unlike standalone structures, in-service bridge beams typically do not require or allow for repairs on all sides. However, full encapsulation was feasible under laboratory conditions. The excess UHPC at the edge of the flange and beam was incidental to the casting procedure adopted in this study. The total volume of UHPC was 0.76 m^3^ (1 yd^3^). Due to delays encountered while sourcing materials, the casting of UHPC occurred eight months after casting the conventional core concrete. A significant time lapse in the casting of dissimilar materials, as often seen in typical repair scenarios spanning 30–50 years, can adversely impact the development of interface shear between the normal-strength concrete substrate and UHPC [30].

Transparent acrylic formwork was assembled around the reduced core to allow the observation of the UHPC flowing around the perimeter of the section. An additional layer of three ϕ16 longitudinal reinforcing steel was provided at the base of the stem. Additional top longitudinal reinforcement, consisting of six ϕ10, was provided above the top flange. To reduce moisture absorption from the UHPC, the core concrete was covered with wet burlap for 24 h before casting the UHPC. The mixing of the UHPC was carried out using multiple shear mixers, which provided a continuous supply of UHPC and prevented the formation of cold joints between successive pours. The UHPC was introduced at the 76.2 mm (3 in) recesses located at the longitudinal ends of the beam and was allowed to fill from the bottom up. After completing the UHPC casting, the specimen was covered with a plastic sheet to prevent moisture loss. The test specimen was cured in ambient laboratory conditions at a temperature of 23±2°C and a humidity of 60±5%.

### 5.4. Test Setup and Instrumentation

**Figure 4:**
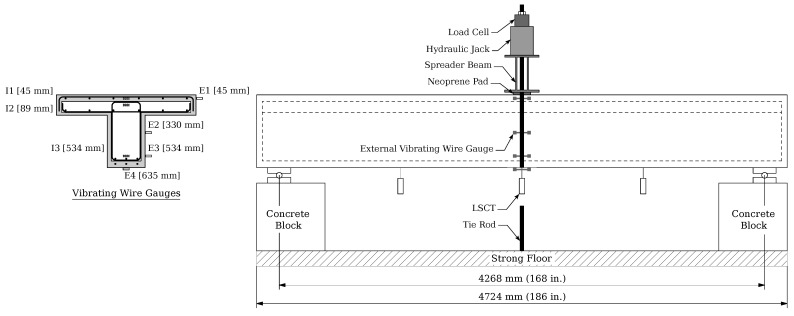
Schematic layout of the ultimate test setup and instrumentation of the T-beam specimen.

The T-beam was subjected to three-point flexural loading during the preloading, cyclic, and ultimate testing phases. Initially, the specimen was placed on a concrete block on top of steel supports, with an effective length of 4268 mm (168 in). A spreader beam, positioned transversely at the mid-span, was used for load application. A neoprene pad measuring 1220 mm × 100 mm with a hardness of 60 durometers was placed between the spreader beam and the flange of the specimen to ensure consistent contact during load application. A 1000 kN hydraulic actuator set was utilized for load application during the cyclic testing. In contrast, the ultimate flexural testing employed a manually operated 1500 kN hydraulic jack set. Figure 4 illustrates the test setup and instrumentation details, while fail-safe concrete blocks were positioned under the specimen during the ultimate testing to prevent potential accidents.

The beam was instrumented with steel strain gauges, load cells, linear strain conversion transducers (LSCTs), and string potentiometers. Additionally, vibrating wire gauges (VWGs) were installed at specific heights at the mid-span section of the beam: ‘Internal VWGs’, designated as I1 to I3, were placed within the beam to measure internal strains, and ‘External VWGs’, labeled E1 to E4, were positioned on the surface of the beam to record external strain measurements. The installation heights of these VWGs are shown in Figure 4. A data acquisition system connected to the instrumentation utilized a 20 Hz sampling frequency during the cyclic and ultimate load testing. Strain gauges were placed on steel reinforcement at mid-span and quarter spans from either end of the supported length, distributed across tension and compression reinforcement. The test results were plotted for select strain gauges at critical sections and load levels.

An abundance of data was received and collected from the flexural tests, which will be discussed in detail in the following sections, focusing on the stiffness degradation, failure mode, load–displacement, internal–external VWGs, and steel strains.

## 6. Cyclic Testing

The cyclic testing was executed using a quasi-static, displacement-controlled loading method. The loading was gradually increased to achieve a cyclic loading range of 133.4 kN (30 kips), fluctuating between minimum and maximum values of 22.2 kN (5 kips) and 155.7 kN (35 kips), respectively. This selected range corresponds to 25% of the flexural strength of the retrofitted specimen, as determined from the moment–curvature analysis. Prior research on the fatigue testing of reinforced UHPC beams has indicated rapid stiffness degradation when subjected to a loading range approximating 50% of the static strength [31,32]. Contrary to this, this study opted for a loading range equivalent to 25% of the flexural strength of the specimen, aiming to monitor stiffness degradation under repeated loading.

**Figure 5:**
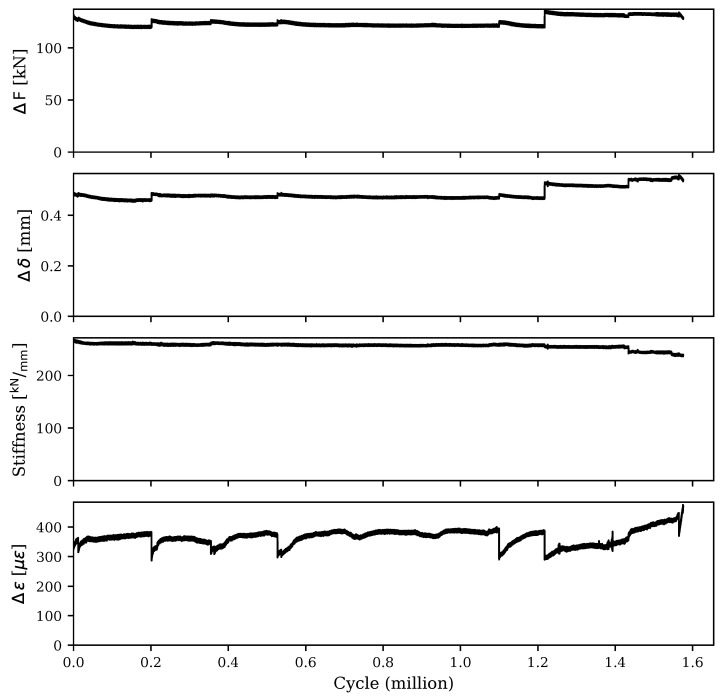
Results of the cyclic testing of the retrofitted specimen.

Figure 5 details the results of the load, mid-span deflection, and strain response ranges throughout the cyclic testing. Initially, the stiffness of the retrofitted specimen was measured at 270 kN/mm. However, this value gradually reduced to approximately 258 kN/mm after 1 million cycles. A slight increase in the loading range was introduced in the latter stages of the cyclic loading, specifically after 1.2 million cycles. This adjustment resulted in a more pronounced stiffness degradation, as shown in Figure 5. It is important to note that the gradual decrease in loading range values can be attributed to the presence of the neoprene pad between the spreader beam and the flange of the specimen. As the cyclic testing progressed, a minor portion of the applied deflections were absorbed by the pad and accumulated as permanent deflections. This accumulation effectively reduced the actual deflection experienced by the specimen, leading to the observed reduction in the loading range. After each pause in the cyclic test, the deflection ranges were recalibrated to achieve the desired loading ranges for the specimen. By the end of the cyclic testing, the specimen had undergone 1.576 million cycles, leading to a final stiffness of 237 kN/mm. This value is equivalent to a 12.22% reduction in the stiffness of the specimen despite the absence of any visible cracks.

Figure 5 also illustrates the strain response range throughout the cyclic testing for the bottom steel reinforcement in the UHPC layer. The strain response range remained relatively consistent, at around 366μϵ. This stability in the strain response is attributed to the relatively low magnitude of the applied load. Since the load was limited to 25% of the flexural strength, it was insufficient to induce crack formation in the web. Consequently, the absence of cracks prevented the reinforcement from experiencing a significant increase in strain, maintaining a steady value well below the yielding strain throughout the testing.

## 7. Ultimate Load Testing

A quasi-static method was employed to apply the load uniformly on top of the specimen at a consistent loading rate. The applied loads, vertical displacement, and strain changes were recorded during testing, accompanied by intermittent visual observations and crack pattern mapping. Testing continued beyond reinforcing bar fractures by completely unloading the specimen and then reapplying the load after adjusting the hydraulic jack stroke to accommodate residual deformations. However, the test was halted after multiple reinforcing bars fractured in the web, and the further continuation of loading was deemed unsafe.

### 7.1. Failure Mode and Load–Displacement Behavior

**Figure 6:**
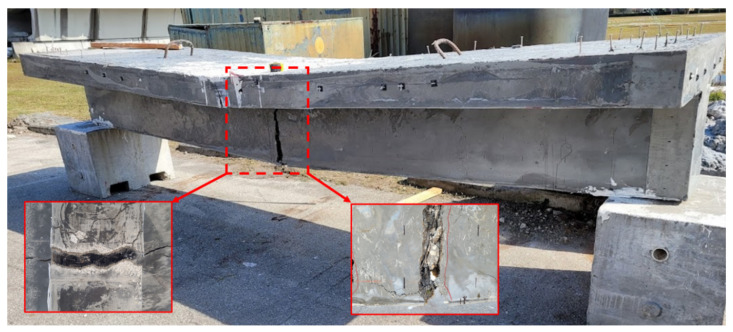
Failure mode of the retrofitted T-beam, illustrating strain localization in the web.

**Figure 7:**
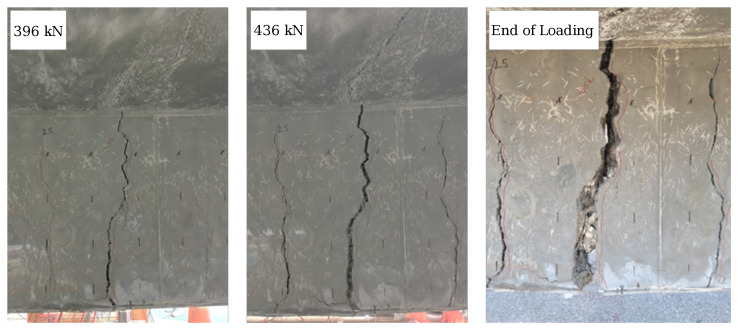
Crack propagation in the web of the retrofitted T-beam at different loading stages.

Figure 6 shows the failure mode of the retrofitted T-beam specimen under the ultimate load setup. The beam behaved monolithically under bending, with successive fractures of reinforcing steel in the web observed upon test termination. Figure 7 shows the crack propagation in the web. The crack development in the beam can be divided into three stages. Stage 1 incorporates three sparsely localized cracks observed at the bottom of the beam, separated over a distance of 686 mm (27 in) at the middle of the tested T-beam. After these localized cracks were initiated, the number of visible cracks did not increase with loading. Stage 2 entails rapidly widening one of the initial cracks at the mid-span after the fiber bridging capacity is exceeded [33,34]. However, the width of the other cracks did not increase significantly with increased loading. Finally, at Stage 3, the three dominant cracks propagated vertically upward to the flange of the beam. At failure, wide cracks were observed in the UHPC layer.

The wide cracks in the web and flange facilitated a visual observation of the state of the concrete core and the interface between UHPC and core concrete. No relative slip was observed between the two materials, indicating the effectiveness of the surface preparation technique in preventing debonding at the crack locations. Although the beam was subjected to significant displacement during testing, the UHPC layer showed no signs of spalling. However, the disintegration of the concrete core was observed at the termination of testing.

**Figure 8:**
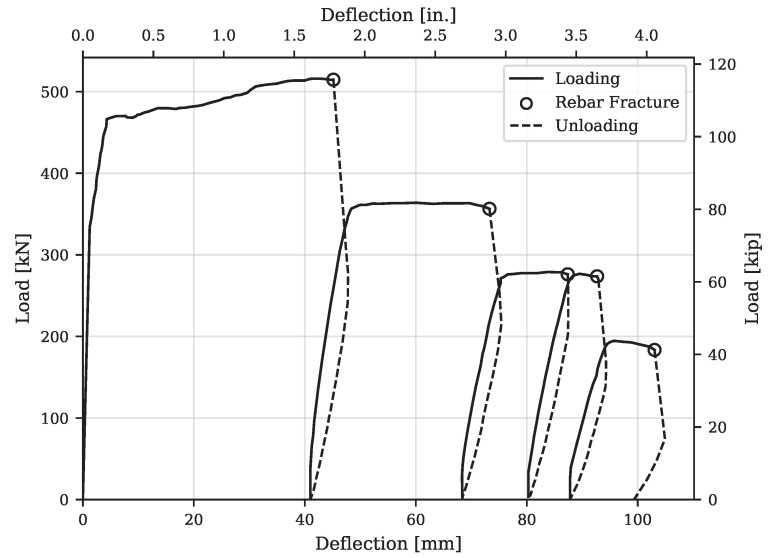
Load–displacement response of the retrofitted T-beam.

Figure 8 shows the mid-span displacement versus load response of the retrofitted specimen. At the initial stages of testing, the retrofitted section exhibited significantly larger flexural stiffness compared to the initially damaged section. As anticipated from the moment–curvature analysis, the initial flexural stiffness of the retrofitted section was approximately 14.9 times greater than that of the damaged section. Consequently, when subjected to a load of 97.9 kN, the mid-span deflection of the retrofitted section was only about 0.4 mm, which is a considerable reduction compared to the 3.8 mm deflection observed in the initially damaged section during the preloading stage. This increase in initial stiffness was attributed to the incorporation of the UHPC layer and the added layer of longitudinal reinforcements, particularly at the bottom of the web inside the UHPC shell.

Under the positive bending moment, the first flexural cracks appeared at the bottom of the web, near the mid-span of the beam. The first cracking was observed when the load reached approximately 335 kN (75.3 kips), corresponding to a displacement of 1.4 mm (0.055 in). After crack formation, a reduction in flexural stiffness was observed. The cracks widened and propagated upward as the load increased to 467 kN (105 kips). Fiber pullout was observed between the macro-cracks in Figure 7, while loud cracking sounds were noted at this stage. The loss of fiber bridging capacity triggered the load reduction. As the load increased, the neutral axis moved upward to the flange, and the macro-cracks on both sides of the web extended to the mid-height of the flange. Further loading did not yield a significant improvement in load-carrying capacity. The width of the macro-cracks in the web increased until a maximum load of 521 kN (117.1 kips). Due to the large flange width and the UHPC overlay, no compressive crushing was observed at the top of the beam throughout the test.

The six tensile reinforcing bars were distributed in the web in two layers, three in the concrete core and three in the UHPC layer, as shown in Figure 1. As expected, the sequence of fracture in the reinforcing bars began with the bars located furthest from the neutral axis. Upon each reinforcing steel’s fracture, there was a sudden drop in load accompanied by a slight increase in mid-span deflection. At this stage, the beam was completely unloaded and subsequently reloaded. The first fracture of reinforcing steel occurred after a maximum load of 521 kN (117.1 kips). Due to the reduction in stiffness, the slope of the reloading branch exhibited a softening behavior. The second and third rebars fractured after the specimen underwent another cycle of unloading and reloading, occurring at loads of 357 kN (80.2 kips) and 276 kN (62.1 kips), respectively. After the complete fracture of the reinforcing steel in the UHPC layer, the rebars in the concrete core also fractured, leading to the termination of the test. A summary of key observations during testing is detailed in Table 1.

### 7.2. Strain Responses

**Figure 9:**
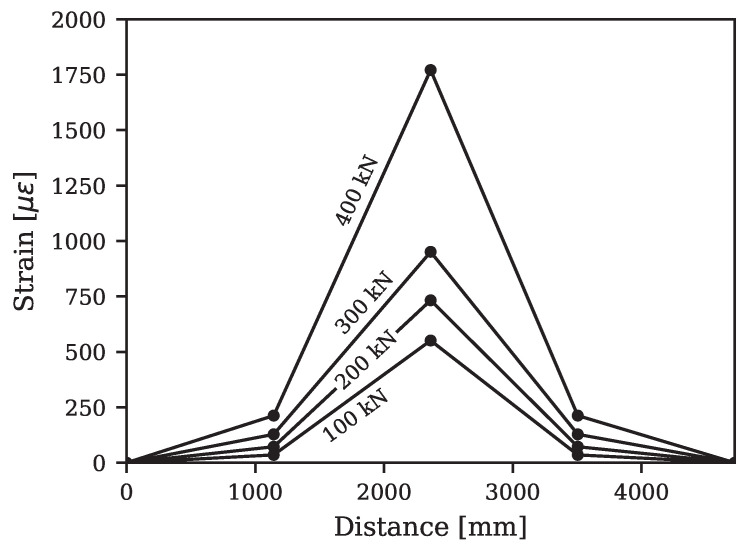
Strain variation in bottom reinforcement of the retrofitted T-beam along its length.

Steel strain gauges were installed on the tension and compression reinforcements at the mid-span and a quarter-span from each end, as shown in Figure 4. The variation in the steel strains for tension reinforcement in the UHPC layer along the length of the beam is plotted in Figure 9. The ordinate of the strain plot shows significantly higher values at the mid-span than those measured at the quarter-span locations. For conventional RC beams, vertical flexural cracks typically appear at the mid-span and then expand horizontally toward the supports as the load increases. However, the UHPC-retrofitted beam exhibited a different behavior, with damage being more localized and predominantly concentrated at the mid-span. This deviation results in a shift from the typical parabolic profile of responses along the length observed in RC beams to a more pronounced mid-span response in the UHPC-retrofitted beam.

**Figure 10:**
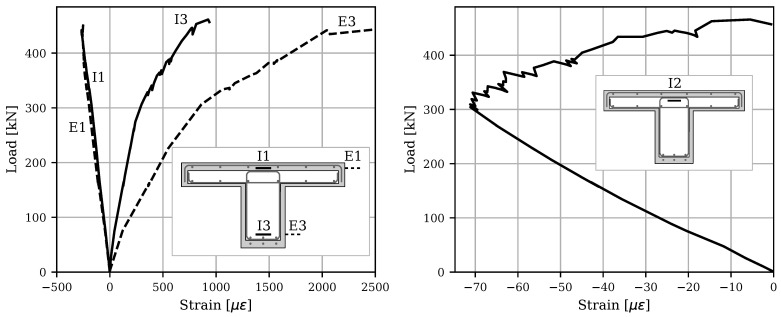
Comparison of the load–strain responses for the internal and external VWGs.

Figure 10 shows the load–strain responses obtained from the internal and external vibrating wire gauges (VWGs) along the depth of the beam. The positive quadrant denotes tensile strains, while the negative quadrant corresponds to compressive strains. Comparing the external and internal VWGs at equal depths in the compressive region reveals similar responses throughout the test. However, the external gauges show significantly higher strain values in the tensile region than their internal counterparts. This difference in strain values between the external and internal gauges becomes more pronounced as the load increases. As highlighted earlier, this discrepancy is mainly attributed to the formation of a localized macro-crack in the UHPC at mid-span that subsequently widens with increased loading. Although mechanical connectors were utilized on the surfaces of the damaged beam to ensure the composite action of the UHPC-retrofitted section, the behavior of the damaged core under flexural loading resembles that of conventional RC beams more closely. Therefore, due to the previous damage to the concrete core and inherent characteristics of conventional concrete, cracks within the center of the damaged core are presumed to be distributed along the length of the beam, contrasting with the localized nature of cracks observed in the UHPC shell (Figure 7). This variance in crack distribution contributes to the observed differences in strain readings, underscoring the complexity of the composite behavior in UHPC-retrofitted structures. In addition, due to the propagation of tension cracks from the web to the flange, the neutral axis also moved upward, resulting in internal gauge I-2 shifting from compressive strain to tensile strain.

## 8. Conclusions and Discussion

This paper presents the results of an artificially damaged concrete T-beam enveloped with a layer of UHPC to investigate the global retrofit methodology and its effectiveness in laboratory conditions. The cyclic testing demonstrated the effectiveness of the retrofit methodology. The retrofitted specimen sustained the applied loads for 1.576 million cycles without any visible cracks. After flexural loading testing was completed, it was concluded that the failure of the beam occurred after multiple tension reinforcing bars were fractured in the T-beam. The UHPC-encased T-beam showed significant improvements in the stiffness and ultimate load capacity, which is attributed to the increase in the moment of inertia of the retrofitted section due to the incorporation of the UHPC shell and the additional layer of longitudinal reinforcements in the web. Moment–curvature analyses performed on damaged and retrofitted T-beam sections showed good agreement against the experimental results regarding flexural stiffness and ultimate capacity. The following are some of the conclusions from the experimental study carried out in this research:

- Despite the absence of visible cracks during cyclic testing, the retrofitted specimen exhibited a 12.22% degradation in stiffness after 1.576 million cycles.
- The strain response in the bottom steel reinforcement in the UHPC layer during cyclic loading remained consistent, averaging around 366μϵ, indicating the effectiveness of the retrofitting method. The relatively low magnitude of the applied load, limited to 25% of the flexural strength, prevented crack formation in the web.
- The failure mode was initiated after localized cracks were observed at the soffit of the web at mid-span. After the loss of fiber-bridging capacity, the crack width increased, and the beam sustained excessive deformation until failure.
- The increase in load-carrying capacity after the formation of localized cracks is attributed to strain hardening in steel reinforcement. With a further increase in load, a series of fractures in steel reinforcing was triggered until the termination of the ultimate test.
- Although a higher repair thickness may be uneconomical, it leads to smaller deformations under service loads. The results showed that fewer macro-cracks developed at higher loads, concentrated at mid-span.
- No relative slip was observed in the specimen. The interface performance was adequate due to sandblasting and the inclusion of steel connectors in the web and flange of the T-beam specimen. No localized fracture of the surrounding concrete was visually observed at the location of the connectors.
- Concrete surface sandblasting can be used as a practical method to provide a good bond between a normal concrete substrate and a thin layer of UHPC.

## Figures and Tables

**Table 1:** Key observations during the ultimate test.

Event	Specimen	Load Level (kN)	Mid-Span Deflection (mm)
Damage simulation under service load	Damaged	97.9	3.8
	Retrofitted	97.9	0.4
Crack observation at bottom	Retrofitted	335	1.4
Reinforcing bar 1 fracture		515	45.2
Reinforcing bar 2 fracture		356	73.3
Reinforcing bar 3 fracture		276	87.4
Reinforcing bar 4 fracture		274	92.7
Reinforcing bar 5 fracture		183	103.0

## Data Availability

The data presented in this study are available upon request from the corresponding author.
